# The genome sequence of the lesser marbled fritillary, *Brenthis ino*, and evidence for a segregating neo-Z chromosome

**DOI:** 10.1093/g3journal/jkac069

**Published:** 2022-03-28

**Authors:** Alexander Mackintosh, Dominik R Laetsch, Tobias Baril, Robert G Foster, Vlad Dincă, Roger Vila, Alexander Hayward, Konrad Lohse

**Affiliations:** 1 Institute of Evolutionary Biology, University of Edinburgh, Edinburgh EH9 3FL, UK; 2 Centre for Ecology and Conservation, University of Exeter, Cornwall TR10 9FE, UK; 3 Edinburgh Genomics, University of Edinburgh, Edinburgh EH9 3FL, UK; 4 Ecology and Genetics Research Unit, University of Oulu, Oulu 90014, Finland; 5 Institut de Biologia Evolutiva (CSIC-Universitat Pompeu Fabra), Barcelona 08003, Spain

**Keywords:** *Brenthis ino*, genome assembly, genome annotation, neo-Z

## Abstract

The lesser marbled fritillary, *Brenthis ino* (Rottemburg, 1775), is a species of Palearctic butterfly. Male *Brenthis ino* individuals have been reported to have between 12 and 14 pairs of chromosomes, a much-reduced chromosome number than is typical in butterflies. Here, we present a chromosome-level genome assembly for *Brenthis ino*, as well as gene and transposable element annotations. The assembly is 411.8 Mb in length with a contig N50 of 9.6 Mb and a scaffold N50 of 29.5 Mb. We also show evidence that the male individual from which we generated HiC data was heterozygous for a neo-Z chromosome, consistent with inheriting 14 chromosomes from one parent and 13 from the other. This genome assembly will be a valuable resource for studying chromosome evolution in Lepidoptera, as well as for comparative and population genomics more generally.

## Introduction

The lesser marbled fritillary, *Brenthis ino* (Rottemburg, 1775), is a species of butterfly in the family Nymphalidae. It has a Palearctic distribution, is widespread in Europe with variance in local abundance, and can be found as far East as Japan and Siberia. It is monovoltine and feeds on plants in the family Rosaceae, including some species in the genera *Filipendula*, *Aruncus*, *Sanguisorba*, and *Rubus*. While most butterflies in the family Nymphalidae, and Lepidoptera more widely, have 31 (or close to 31) pairs of chromosomes (de Vos *et al.* 2020), *B. ino*, along with its sister species *B. daphne* (Denis and Schiffermüller, 1775), has an unusually low chromosome count. Federley (2010) reported male haploid chromosome numbers of 12 and 13 for individuals collected in Finland, consistent with segregating chromosomal fissions or fusions in the population. However, other males sampled in Finland and Sweden consistently displayed 13 chromosome pairs (Saitoh 1987, 1991). In Japan, where the subspecies *B. ino mashuensis* (Kono, 1931) and *B. ino tigroides* (Fruhstorfer, 1907) are found, a male chromosome number of 14 has been consistently observed (Maeki and Makino 1953; Saitoh *et al.* 1989).

Currently, there are no genome assemblies for species in the genus *Brenthis* and information about chromosome evolution in the genus is confined to cytological data. Here, we present a chromosome-level genome assembly of *B. ino* as well as gene and transposable element (TE) annotations. We also show that one of the individuals we sampled was heterozygous for a neo-Z chromosome, consistent with there being karyotypic variation within the Spanish population from which we sampled.

## Materials and methods

### Sampling

Three individuals were collected by hand netting in Somiedo, Braña de Mumian, Asturias, Spain (SO_BI_364, SO_BI_375, SO_BI_376) and one in Larche, Alpes-de-Haute-Provence, France (FR_BI_1497, RVcoll12O846) (Supplementary Table 1). Spanish individuals were flash frozen in a liquid nitrogen dry shipper. The French specimen was dried and, after some days, stored in ethanol at −20°C.

### Sequencing

High molecular weight (HMW) DNA was extracted from the thorax of a flash-frozen individual (SO_BI_364) using a salting out extraction protocol. In brief, tissue was homogenized in cell lysis buffer using a micro-pestle and then incubated with Proteinase K overnight at 56°C, followed by a further 1-h incubation at 37°C with RNase A, before precipitating and discarding proteins. Finally, DNA was precipitated using isopropanol and the resulting pellet was washed with ethanol.

Edinburgh Genomics (EG) generated a SMRTbell sequencing library from the HMW DNA, which was sequenced on 3 SMRT cells on a Sequel I instrument to generate 28.4 Gb of Pacbio continuous long read (CLR) sequence data. From the same HMW DNA extraction, EG also generated a TruSeq library (350-bp insert) and 33.5 Gb of Illumina whole genome (WGS) paired-end reads on a Novaseq 6000. Pacbio and Illumina protocols were followed for library preparation, QC and sequencing.

A second individual (SO_BI_375) was used for chromatin conformation capture (HiC) sequencing. The HiC reaction was done using an Arima-HiC kit, following the manufacturer’s instructions for flash-frozen animal tissue. The NEBNext Ultra II library was sequenced on an Illumina MiSeq at EG, generating 4.8 Gb of paired-end reads.

Illumina WGS paired-end reads were also generated for the same individual used for HiC sequencing (SO_BI_375) as well as the French female individual (FR_BI_1497) that did not contribute to the assembly.

Paired-end RNA-seq data (for individual SO_BI_376) were previously generated and analyzed by Ebdon *et al.* (2021) (ENA experiment accession ERX5086186).

### Genome assembly

Illumina WGS, RNA-seq, and HiC reads were adapter and quality trimmed with fastp v0.2.1 (Chen *et al.* 2018).

The Pacbio reads were assembled with Nextdenovo v2.4.0 (Hu 2021) using default parameters. Contigs were polished twice by aligning Illumina WGS reads and correcting consensus errors with HAPO-G v1.1 (Aury and Istace 2021). Contigs belonging to nontarget organisms were identified using blobtools v1.1.1 (Laetsch and Blaxter 2017) and subsequently removed. Duplicated regions (haplotigs and overlaps) were identified and removed with purge_dups v1.2.5 (Guan *et al.* 2020). Mapping of Pacbio reads and Illumina WGS reads for the above steps was performed with minimap2 v2.17 and bwa-mem v0.7.17, respectively (Li 2013, 2018).

The trimmed HiC reads were aligned to the contig-level assembly with Juicer v1.6 (Durand *et al.* 2016). Scaffolding was performed with 3D-DNA v180922 (Dudchenko *et al.* 2017). The initial scaffolding generated by 3D-DNA was manually partitioned into chromosomes and misassembly corrected with Juicebox v1.11.08 (Robinson *et al.* 2018).

A k-mer spectrum, with *k *=* *21 and a maximum counter value of 10^7^, was generated using KMC v3.1.1 (Kokot *et al.* 2017) and genome size was estimated from the spectrum using Genomescope v2.0 (Ranallo-Benavidez *et al.* 2020).

Gene completeness was evaluated using BUSCO v5.2.2 with the insecta_odb10 dataset (n = 1367) (Manni *et al.* 2021). Kmer QV was calculated using Merqury v1.3 (Rhie *et al.* 2020).

The mitochondrial genome was assembled and annotated using the Mitofinder pipeline v1.4 (Allio *et al.* 2020). Illumina WGS reads from SO_BI_364 were assembled with metaSPAdes v3.14.1 (Nurk *et al.* 2017) and tRNAs were annotated with MiTFi (Jühling *et al.* 2012).

### Karyotype analysis

After scaffolding, chromosomes 11 and 13 displayed an intermediate HiC contact map pattern, suggesting a potential fusion of the chromosomes in one of the haplotypes.

To investigate this further we generated haplotype-specific HiC maps for chromosomes 11 and 13. First, we created a version of the assembly where chromosomes 11 and 13 were scaffolded together. WGS and HiC reads (from SO_BI_375) were mapped to this assembly with bwa-mem v0.7.17. Alignments were deduplicated with sambamba v0.6.6 (Tarasov *et al.* 2015). Heterozygous variants were called from the WGS alignments with freebayes v1.3.2-dirty (Garrison and Marth 2012). Variants were then normalized with bcftools v1.8 (Danecek *et al.* 2021) and decomposed with vcfallelicprimitives (Garrison *et al.* 2021). Normalization involves left-aligning variants and ensuring that they are represented parsimoniously. Decomposition is the splitting up of MNPs and complex variants into multiple SNPs and/or indels. Variants were filtered for coverage (>7 and <56 reads) with bcftools. The remaining SNPs were phased using HAPCUT2 v1.3.3 with both the WGS and HiC alignments as input (Edge *et al.* 2017).

We developed a tool (chomper.py, see *Data Availability*), which uses the phased SNPs from HAPCUT2 to partition aligned HiC reads by haplotype. For any read pair whose alignment encompasses at least one phased SNP, we can ask whether the alleles in the read are associated with haplotype 1 or 2. If a read pair contains alleles exclusively associated with one haplotype, then it is assigned to that haplotype-specific read set. If it instead contains alleles associated with both haplotypes, then it is discarded. Haplotype-specific HiC read sets were then aligned back to the original assembly with Juicer and visualized with HiC_view.py (parameters -b 250 -s 10, see *Data Availability*).

To identify the Z chromosome, one male (SO_BI_364) and one female (FR_BI_1497) individual were mapped to the assembly with bwa-mem v0.7.17 and median, window-wise coverage was calculated using mosdepth v0.3.2 (Pedersen and Quinlan 2018).

### Synteny comparison

Synteny in the *B. ino* genome was compared to synteny in another Nymphalid genome, *Melitaea cinxia* (GCA_905220565.1; Vila *et al.* 2021). A total of 5178 lepidoptera_obd10 BUSCO genes were identified in both assemblies using BUSCO v5.2.2. The positions of these genes in both assemblies were visualized using busco2synteny.py (see *Data Availability*).

### Genome annotation

The Illumina RNA-seq reads were mapped to the assembly with HISAT2 v2.1.0 (Kim *et al.* 2019). The softmasked assembly and RNA-seq alignments were used for gene prediction with braker2.1.5 (Stanke *et al.* 2006, 2008; Li *et al.* 2009; Barnett *et al.* 2011; Lomsadze *et al.* 2014; Buchfink *et al.* 2015; Hoff *et al.* 2016, 2019). Gene annotation statistics were calculated with GenomeTools v1.6.1 (Gremme *et al.*, 2013).

TEs were annotated using the Earl Grey TE annotation pipeline (https://github.com/TobyBaril/EarlGrey, Baril *et al.* 2021). Briefly, known repeats were masked with RepeatMasker v4.1.2 (Smit *et al.* 2015) using the Lepidoptera library from RepBase v23.08 and Dfam release 3.3 (Jurka *et al.* 2005; Hubley *et al.* 2016). Following this, a de novo repeat library was constructed using RepeatModeler2 v2.0.2 (Flynn *et al.* 2020) with RECON v1.08 and RepeatScout v1.0.6. Subsequently, Earl Grey generated maximum-length consensus sequences for the de novo sequences identified by RepeatModeler2 using an automated version of the “BLAST, Extract, Extend” process, as previously described (Platt *et al.* 2016). The resulting de novo repeat library was combined with the RepBase and Dfam libraries used in the initial masking step to annotate repetitive elements using RepeatMasker. Full-length LTR elements were identified using LTR_Finder v1.07 with the LTR_Finder parallel wrapper (Xu and Wang 2007; Ou and Jiang 2019). Final TE annotations were defragmented and refined using a loose merge in RepeatCraft (-loose), followed by maintaining the longest of any overlapping annotations with MGkit v0.4.1 (filter-gff -c length -a length) (Rubino and Creevey 2014; Wong and Simakov 2019). Finally, all repeats <100 bp in length were removed before final TE quantification to decrease spurious hits.

Following gene annotation, gene flanks were defined as regions that were ≤20-kb upstream and downstream of genes. We expect these regions to be enriched for regulatory sequences, including both proximal promoters and distal elements. We define regions as intergenic if they are neither genic (start/stop codons, exons, and introns) nor gene flanks. Bedtools intersect v2.27.1 (Quinlan and Hall 2010) was used to determine overlap (-wao) between TEs and genomic features. Following this, quantification and plotting was performed in R, using the tidyverse package (Wickham *et al.* 2019; RStudio Team 2020; R Core Team 2021).

### Estimating heterozygosity

To estimate heterozygosity, WGS reads were mapped to the assembly with bwa-mem v0.7.17 and variants were called with freebayes v1.3.2-dirty. Variant calls were normalized with bcftools v1.8 and decomposed using vcfallelicprimitives (for an explanation of these terms, see *Karyotype* *Analysis*). Callable sites, where coverage was >7 and less than twice the sample mean, were identified using mosdepth v0.3.2. Fourfold-degenerate sites, where all possible nucleotide substitutions have no effect on the amino acid sequence, were identified using partition_cds.py (see *Data Availability*). Biallelic SNPs within callable fourfold-degenerate sites were counted using bedtools v2.30.0. To calculate heterozygosity, SNP counts were divided by the total number of callable fourfold-degenerate sites for each individual.

## Results

### Genome assembly

**Fig. 1.**
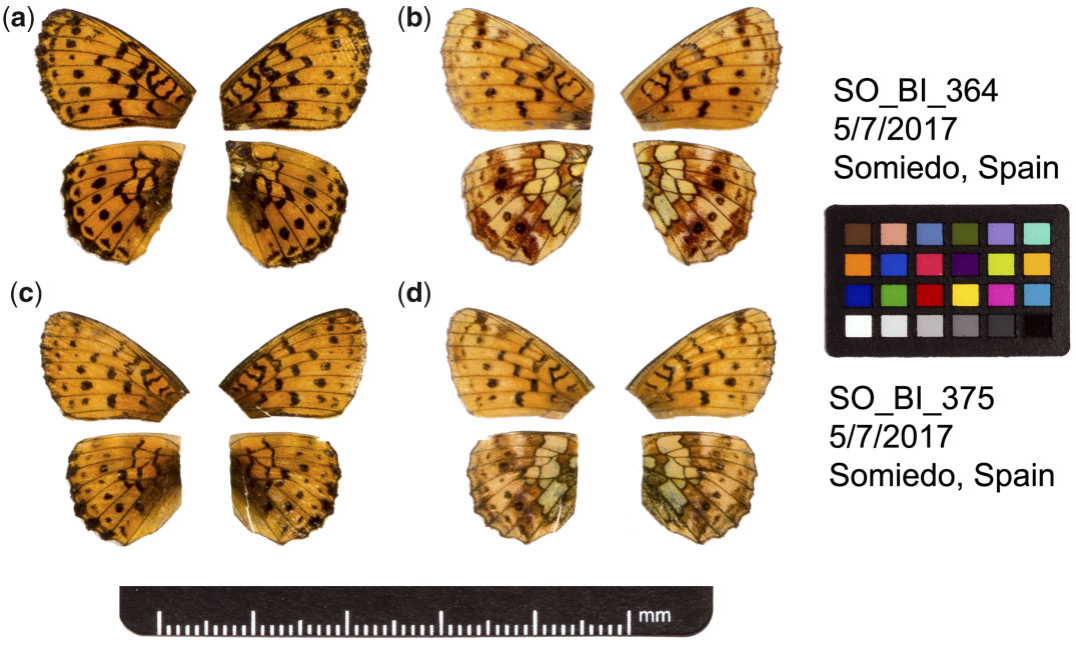
Fore and hind wings of the two *B. ino* individuals used to generate the genome sequence. a) Dorsal and b) ventral surface view of wings of specimen SO_BI_364, used to generate Pacbio and Illumina WGS reads. c) Dorsal and d) ventral surface view of wings of specimen SO_BI_375, used to generate HiC reads.

We sequenced and assembled the genome of a male *B.* *ino* individual collected in Asturias, Spain (SO_BI_364, Fig. 1, a and b). We generated 69.0x and 81.2x coverage of Pacbio CLR and Illumina WGS reads, respectively. The initial assembly consisted of 119 contigs and had a total length of 411.8 Mb, which is consistent with the kmer-based estimate of haploid genome size of 414.0 Mb (Supplementary Fig. 1). HiC reads (11.7x coverage) from a male specimen collected at the same locality (SO_BI_375, Fig. 1, c and d) were used to scaffold the contigs into 14 chromosome-level sequences. These scaffolds range in size from 21.9 to 43.0 Mb and encompass 99.7% of the assembly. The contig and scaffold N50 of the assembly is 9.6 and 29.5 Mb, respectively.

The BUSCO score of the assembly is 99.0% (S: 98.6%, D: 0.4%, F: 0.3%, M: 0.7%), suggesting that the assembly is missing very few single-copy insect orthologues and has little duplication. The estimated mean Phred quality score of the consensus sequence is 39.85.

We assembled and annotated a circular mitochondrial genome of 15,180 bases with 13 protein coding genes, 22 tRNAs, and 2 rRNAs. The cytochrome oxidase subunit 1 (COI) nucleotide sequence has 99.85% identity (657/658 b) with a previously published COI sequence from a *B. ino* individual collected in Castilla y León, Spain (GenBank accession MN144802, Dapporto *et al.* 2019).

### Evidence for a segregating neo-Z chromosome

**Fig. 2.**
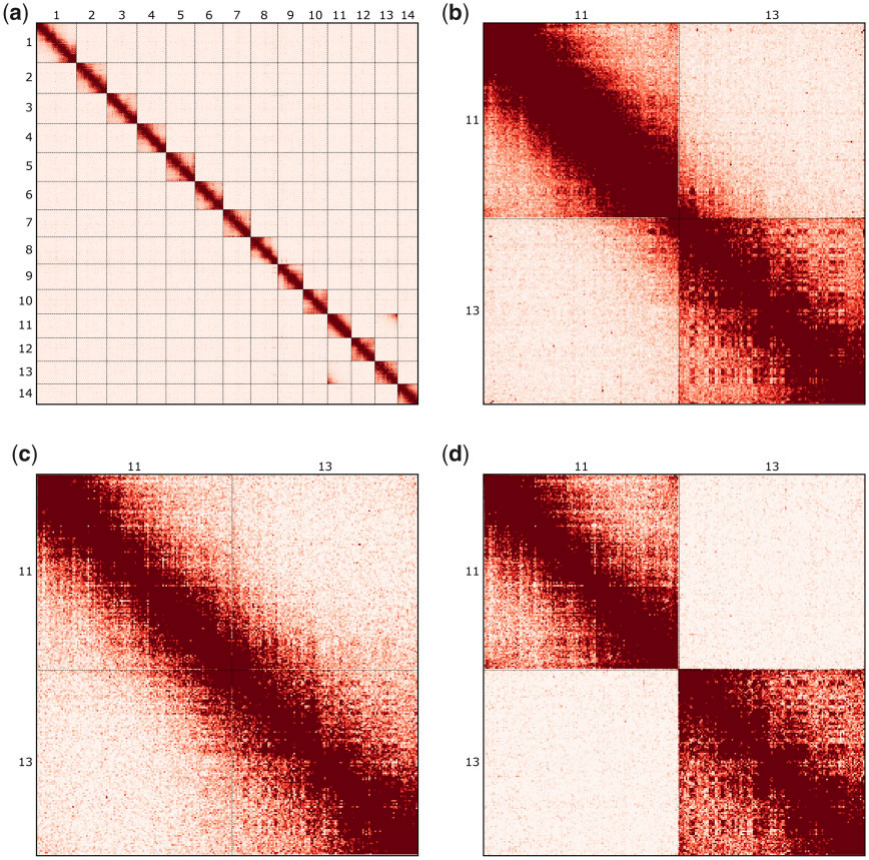
HiC contact heatmaps for the assembly of *B. ino*. a) HiC contacts across all 14 chromosomes (HiC_view params: -b 2500 -s 25). b) Contacts across chromosomes 11 and 13, with both chromosomes in the reverse orientation (HiC_view params: -b 250 -s 30). c) The same as in (b) but restricted to HiC reads containing alleles exclusively associated with haplotype 1 (HiC_view params: -b 250 -s 10). d) The same as in (c) but associated with haplotype 2 rather than 1 (HiC_view params: -b 250 -s 10).

While the HiC data support the scaffolding of 14 chromosome-level sequences (hereafter simply referred to as chromosomes), there is an excess of HiC contacts between chromosomes 11 and 13 (Fig. 2a). This excess is not distributed evenly over the two chromosomes and is instead concentrated at one of the four possible junctions (Fig. 2b), supporting the scaffolding of these two chromosomes in a specific orientation. However, while the number of HiC contacts between chromosomes 11 and 13 exceeds what we see between any other pair of chromosomes, it is below what we typically observe within chromosomes in this dataset (Supplementary Fig. 2), making it unclear whether chromosomes 11 and 13 are fused and should be scaffolded together.

We tested whether the HiC contacts between chromosomes 11 and 13 are haplotype specific, as this would result in half the number of contacts, and so could explain the reduced frequency (Supplementary Fig. 2). Haplotype-specific HiC maps (see *Materials and* *Methods*) confirm that HiC contacts between chromosomes 11 and 13 are almost entirely limited to one haplotype (Fig. 2, c and d) and the proportions of haplotype-specific reads (49.6% and 50.4% of partitioned reads support haplotypes 1 and 2, respectively) are consistent with these chromosomes being fused in one haplotype but not the other.

We identified chromosome 11 as the Z-chromosome in *B. ino*: the female individual (Supplementary Fig. 3) has half coverage for this chromosome, whereas the male used for assembly has full coverage (Supplementary Fig. 4). By contrast, chromosome 13 has full coverage in both males and females (Supplementary Fig. 4), consistent with the expectation for autosomal chromosomes (although see *Discussion*). As one of these chromosomes is Z-linked, while the other has autosomal patterns of sex-specific coverage, we conclude that the individual from which we generated the HiC library must be heterozygous for a Z-autosome fusion, i.e. a neo-Z chromosome.

The Pacbio reads, which were generated from SO_BI_364 rather than SO_BI_375, do not span the gap between chromosomes 11 and 13. However, it is still possible that SO_BI_364 does possess a copy of the neo-Z chromosome, if the fusion point is within a region of the genome that is too repetitive to be assembled and the gap is too large for successful chimeric alignment. It is therefore uncertain whether only SO_BI_375 possesses a copy of the neo-Z or if SO_BI_364 does as well.

### Synteny

**Fig. 3.**
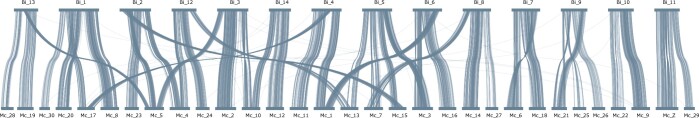
A synteny comparison between *B. ino* (top) and *M. cinxia* (bottom). Each line connects the same BUSCO gene in either genome assembly. Chromosomes are ordered to minimize the number of lines that cross one another. The correspondence between *M. cinxia* and *B. ino* chromosomes can only be explained by chromosome fusions alongside fissions and/or reciprocal translocations.

We expect that the *B. ino* genome has been shaped by many chromosome fusions because it has a much lower chromosome number than other Nymphalid butterflies. A pairwise comparison of synteny between *B. ino* and *M.* *cinxia* shows that all *B. ino* chromosomes contain genes from multiple *M. cinxia* chromosomes (Fig. 3). In addition, nine *M. cinxia* chromosomes have genes distributed over multiple *B. ino* chromosomes (Fig. 3). Because *M. cinxia* possesses the ancestral karyotype of Nymphalid butterflies (Ahola *et al.* 2014), the differences in synteny observed in Fig. 3 are all the result of rearrangements on the lineage leading to *B. ino*. These patterns of synteny therefore show that chromosome fusions, alongside fissions and/or reciprocal translocations, have shaped the *B. ino* genome.

### Genome annotation

We annotated 16,844 protein coding genes. Given this annotation, we estimate that 33.5% of the genome assembly is intronic and 5.6% exonic. Chromosomes display some variation in gene density; chromosome 14, the shortest and most gene poor, is 32.8% genic whereas chromosome 11 (the Z) is 47.7% genic. Across the annotation, the median length of genes, introns, and exons is 4084, 616, and 148b, respectively (Supplementary Fig. 5).

**Fig. 4.**
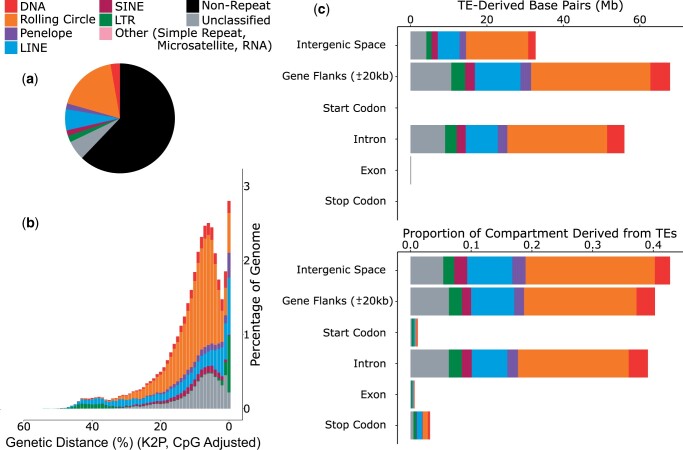
TEs within the genome assembly of *B. ino*. a) The proportion of the assembly comprised of the main TE classifications, as represented by the colors in the key. b) A repeat landscape plot illustrating the proportion of repeats in the genome at different genetic distances (%) to their respective RepeatModeler consensus sequence. Greater similarity to consensus (i.e. lower genetic distance) is suggestive of recent activity. c) The abundance of TEs in different partitions of the genome, shown in bases and as a proportion of the partition.

TEs compromise 37.9% of the genome (Supplementary Table 2 and Fig. 4a). Most TE activity appears to be relatively recent, as a large proportion of repeats exhibit a low genetic distance from their respective consensus sequences (Fig. 4b). The genome contains all major TE types (Supplementary Table 2). Rolling circle elements, also known as helitrons, appear to have been the most successful progenitors within the genome, accounting for 17.8% of total genome length, and ∼ 47% of total TE content (Supplementary Table 2). There is also evidence of very recent activity in LINEs and LTR elements, with a sharp increase in the number of identified elements with very low genetic distance to their consensus sequences (Fig. 4b). The reasons for the bursts in LINEs and LTRs are unknown, although the likely recent age of these insertions is consistent with recent host colonization, potentially via horizontal transposon transfer from another host genome (Gilbert *et al.* 2010; Wallau *et al.* 2012; Ivancevic *et al.* 2018).

Considering all TE classifications, most TEs are found outside of genes (Fig. 4c). Gene flanks and introns have a similar density of TEs, whereas intergenic space has a slightly higher density (Fig. 4c). Exons are largely devoid of TE sequence, with only 0.7% of exonic sequences consisting of TEs. This is to be expected given the likely detrimental effects of TE insertions in host exons (Sultana *et al.* 2017; Bourque *et al.* 2018). The most abundant TEs in the genome, rolling circle elements, comprise 21.3% of intergenic space, ∼18% of gene flanks and intronic regions, and just 0.1% of exonic regions (Fig. 4c).

Satellite repeats are found immediately adjacent to the putative neo-Z fusion point. Chromosome 11 starts with a 5.8-kb array of repeats (RND-5_FAMILY-919) and chromosome 13 ends in a 10.9-kb array (RND-6_FAMILY-6270). The array on chromosome 11 consists of repeat units of ∼110 bases, whereas the array on chromosome 13 has larger repeat units of ∼325 bases. We conclude that, due to a lack of similarity, these repeats are unlikely to have facilitated a nonhomologous recombination event that led to the neo-Z fusion.

## Discussion

We have resolved the sequences of 14 *B.* *ino* chromosomes: 13 autosomes and the Z sex-chromosome. The number of chromosomes in the assembly is higher than previously reported for *B. ino* in Europe (Saitoh 1987, 1991; Federley 2010), but equal to counts reported for this species in Japan (Maeki and Makino 1953; Saitoh *et al.* 1989). We note that previous karyotype data from Europe were all from Scandinavian samples, whereas the individuals contributing to the assembly were collected in Spain. Scandinavian populations of *B. ino* may therefore have a high frequency of the neo-Z fusion that we report or other chromosome fusions that are not identifiable in our data.

We have interpreted the excess of HiC contacts between chromosomes 11 and 13, as well as the stark contrast in haplotype-specific HiC maps, as strong evidence for a segregating neo-Z chromosome. Lab contamination from a closely related—but karyotypically divergent—species is not a plausible alternative explanation given that the haplotype partitioned HiC reads are approximately equal in frequency (see *Results*). We can also rule out the possibility that we sampled an admixed individual, for example, an F1 between *B. ino* and its sister species *B. daphne*, and that the neo-Z is fixed in one species but absent in the other. Both species are present in Northern Spain, so sampling an F1 is possible, at least in principle. However, if SO_BI_375 were a recent hybrid, we would expect its heterozygosity to be considerably elevated compared to other *B. ino* individuals, which is not the case: heterozygosity at autosomal fourfold degenerate sites for SO_BI_375, SO_BI_364, and FR_BI_1497, is 0.0108, 0.0106, and 0.0100, respectively, and in all cases is far lower than we would expect for an F1 between *B. ino* and *B. daphne* (∼0.025, Ebdon *et al.* 2021).

Because we have only observed evidence for the neo-Z in one individual, we do not know its frequency in the wider *B. ino* population. This rearrangement could be restricted to certain populations, or it may have evolved so recently that it is only found in a small number of closely related individuals. One way to estimate the frequency of the neo-Z would be to test whether any females have half the normalized coverage over both chromosomes 11 and 13, which would be consistent with a single copy of the neo-Z (chromosomes 11 and 13 fused together), a W chromosome, but no additional copy of chromosome 13. However, if chromosome 13 is yet to evolve a dosage compensation mechanism, females carrying the neo-Z may only be viable with two copies of the autosomal sequence. Under this scenario, the female coverage seen in Supplementary Fig. 4 is consistent with both presence or absence of the neo-Z chromosome. Population level cytological or HiC data would be required to estimate the frequency of the neo-Z and understand its evolutionary history.

While we have mainly focused on karyotypic variation within a single individual, we have also shown that the *B. ino* genome has a complex rearrangement history that includes many fusions as well as fissions and/or reciprocal translocations (Fig. 3). The assembly therefore provides an opportunity to test the causes and consequences of chromosome rearrangements more widely. In addition, the assembly will enable population genomic studies in the genus *Brenthis*, expanding on previous reference-free analyses (Pazhenkova and Lukhtanov 2019; Ebdon *et al.* 2021). More generally, it adds to a growing number of high-quality resources for comparative genomics in the Lepidoptera.

## Data availability

Supplementary Table 1 contains the metadata for the four individuals used for this project. The genome assembly, gene annotation, and raw sequence data can be found at the European Nucleotide Archive under project accession PRJEB49202. The scripts used for analyzing HiC data (chomper.py and HiC_view.py), the script used for calculating site degeneracy (partition_cds.py), and the script used for visualizing synteny (busco2synteny.py) can be found at the following github repository: https://github.com/A-J-F-Mackintosh/Mackintosh_et_al_2022_Bino. The mitochondrial genome sequence and the TE annotation can be found at the same repository.

Supplemental material is available at *G3* online.

## Supplementary Material

jkac069_Supplementary_Data
