# Supplementary material for: The genome sequence of the lesser marbled fritillary, Brenthis ino, and evidence for a segregating neo-Z chromosome
Source: G3 (Bethesda). 2022 Mar 28;12(6):jkac069. doi: 10.1093/g3journal/jkac069 (PMC9157119; doi:10.1093/g3journal/jkac069)
Supplement: jkac069_Supplementary_Data [file jkac069_supplementary_data.pdf]

Supplementary Materials

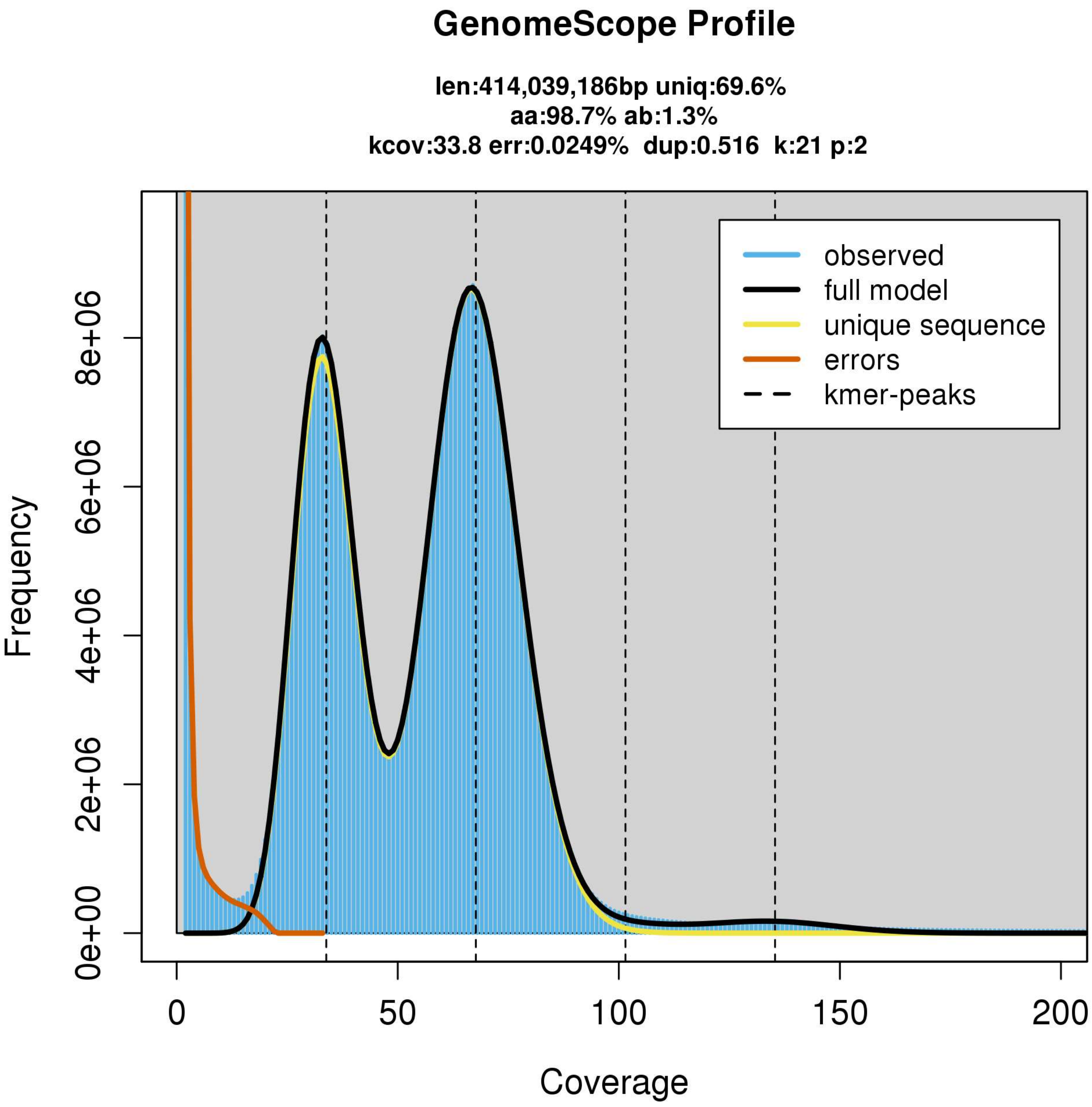

Figure S1 Kmer spectrum and Genomescope parameter estimates for SO_BI_364.

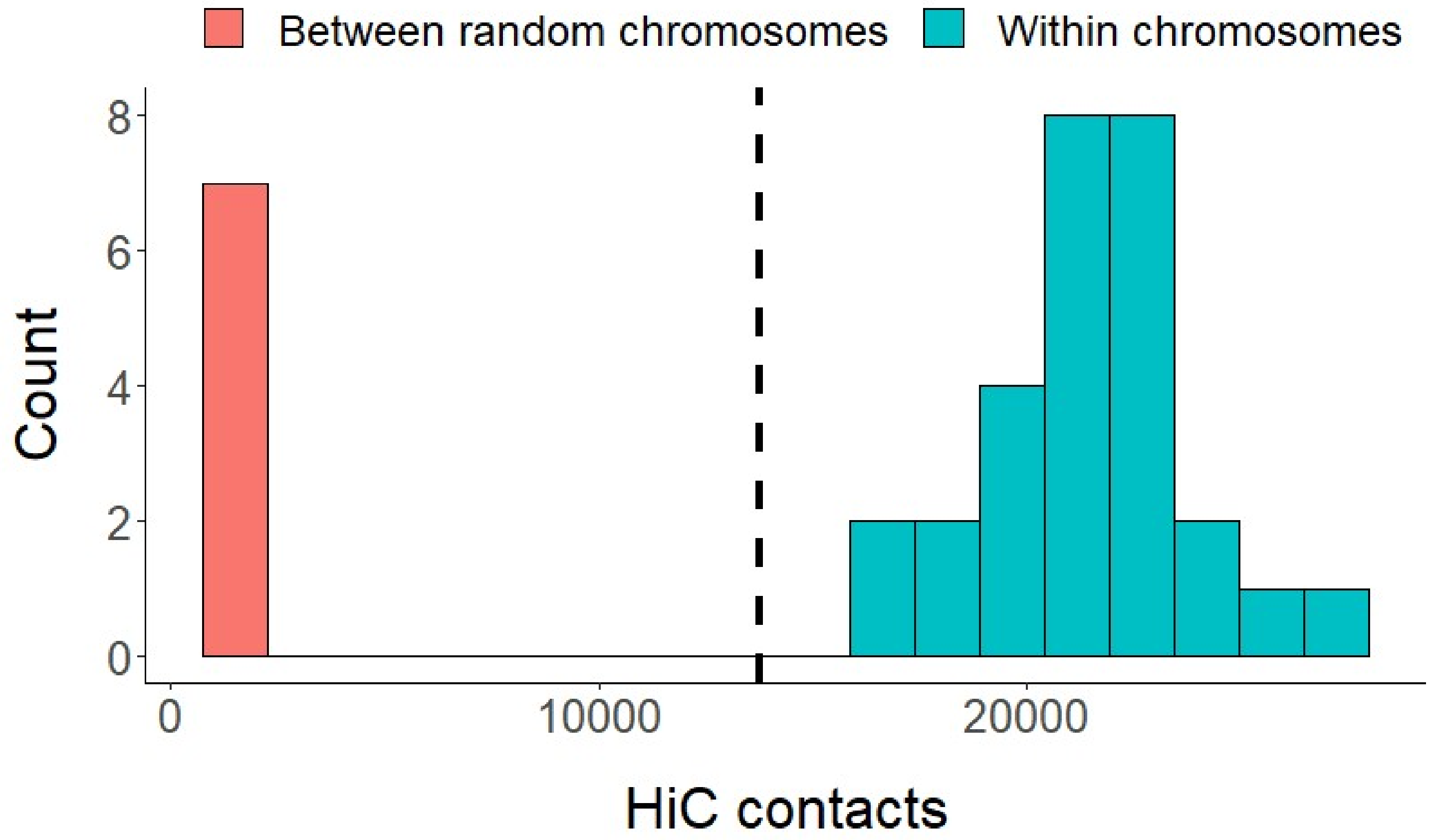

Figure S2 A histogram of HiC contact frequency within and between chromosomes. In red, the number of contacts spanning fusion points of randomly fused chromosome pairs, where either read is within 5Mb of the fusion point and each chromosome has been randomly fused once. In blue, the number of contacts spanning arbitrary points within chromosomes, where reads are again within 5Mb and two independent points are sampled per chromosome. The dashed line represents the number of contacts spanning the putative neo-Z fusion between chromosomes 11 and 13. The frequency of contacts supporting the neo-Z is consistent with heterozygosity.

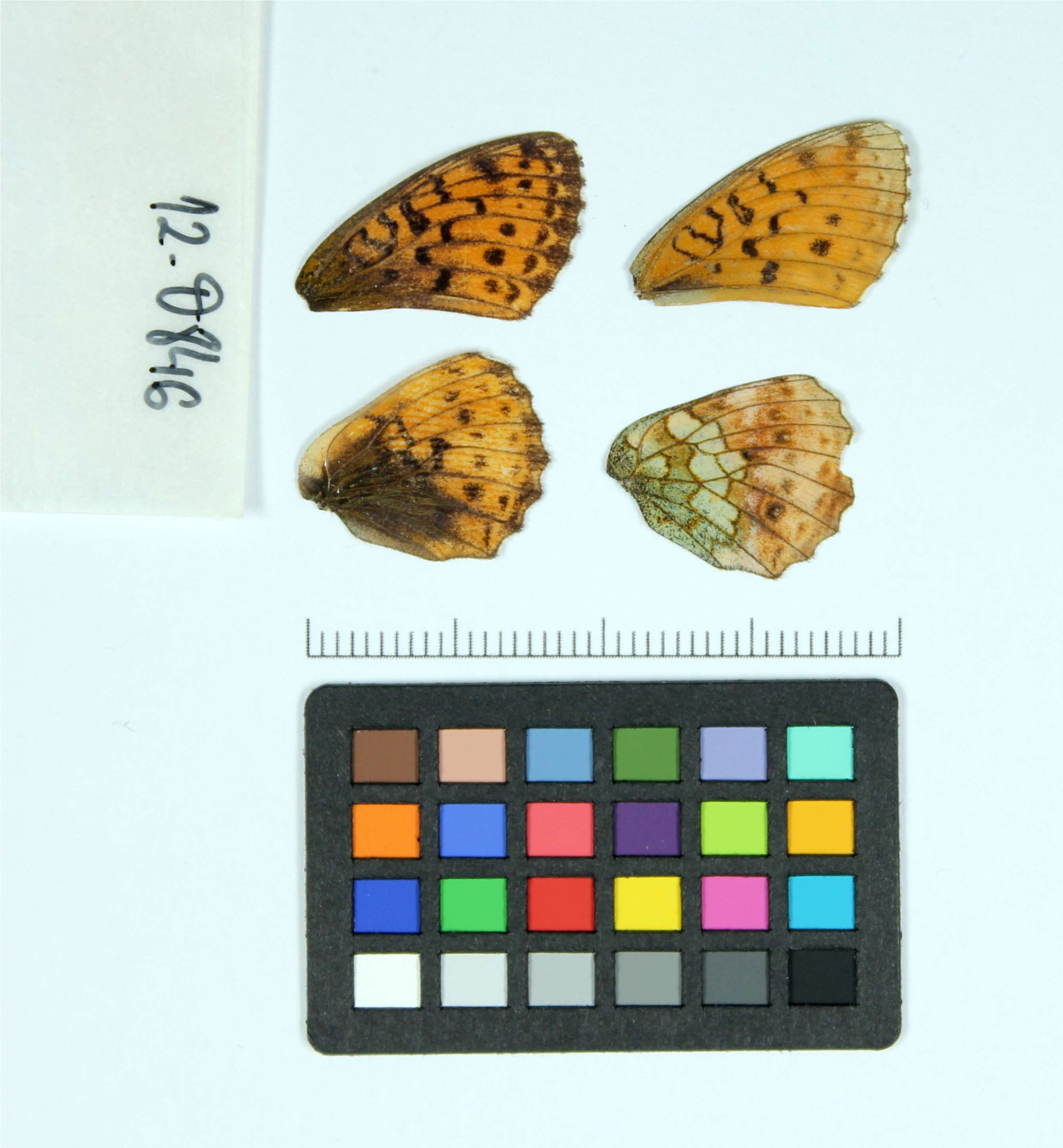

Figure S3 Wings of the female specimen FR_BI_1497. Top-left: dorsal forewing. Top-right: ventral forewing. Bottom-left: dorsal hindwing. Bottom-right: ventral hindwing

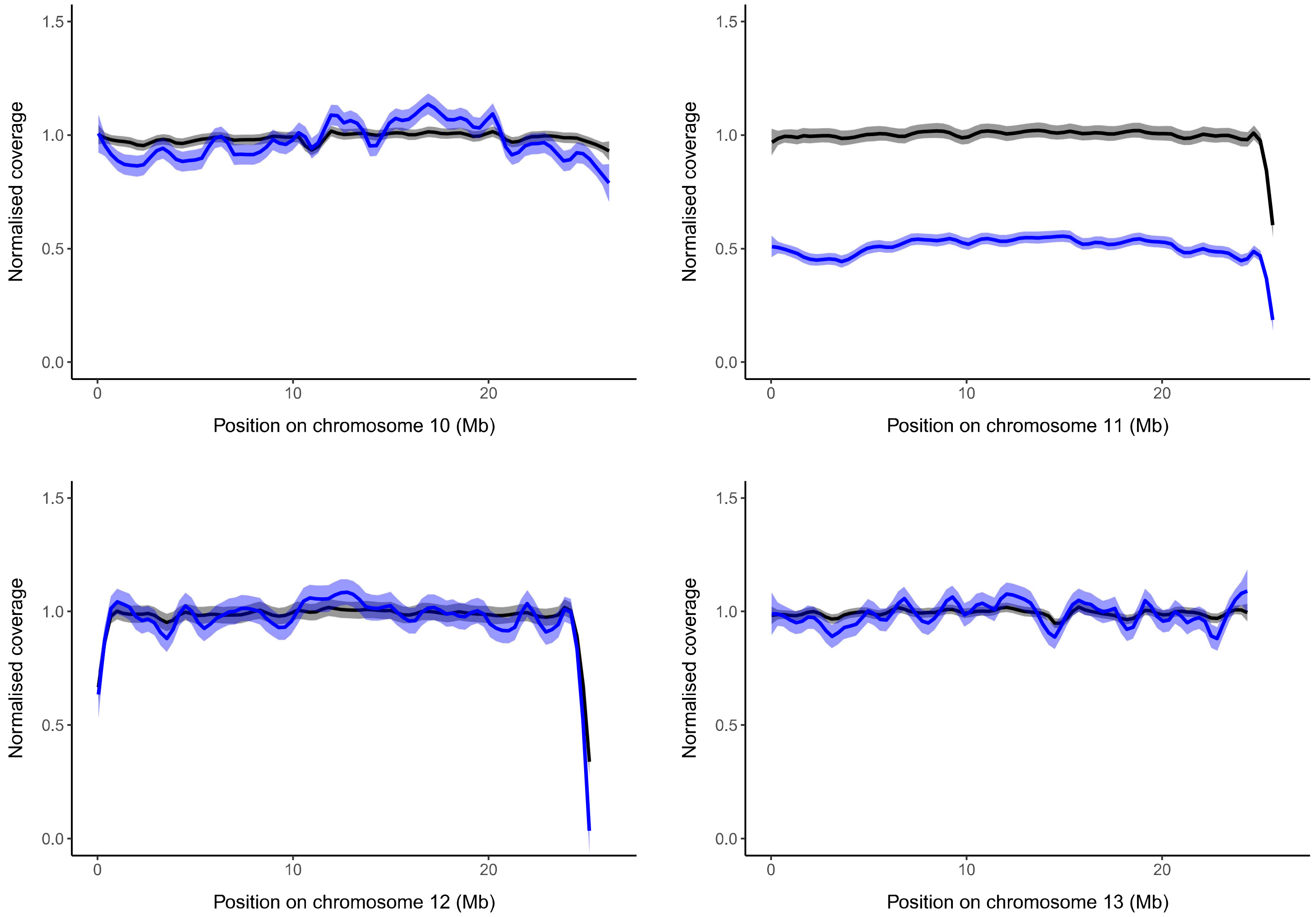

Figure S4 Normalised coverage on four chromosomes. The black line represents normalised coverage of WGS reads from the male used for genome assembly (SO_BI_364), while the blue line is normalised coverage of WGS reads from a female individual (FR_BI_1497). On chromosome 11 the male has full coverage whereas the female has half coverage, consistent with expectations for Z-linked chromosomes in Lepidoptera. On the other three chromosomes, as well as the ten not shown, both individuals have full normalised coverage.

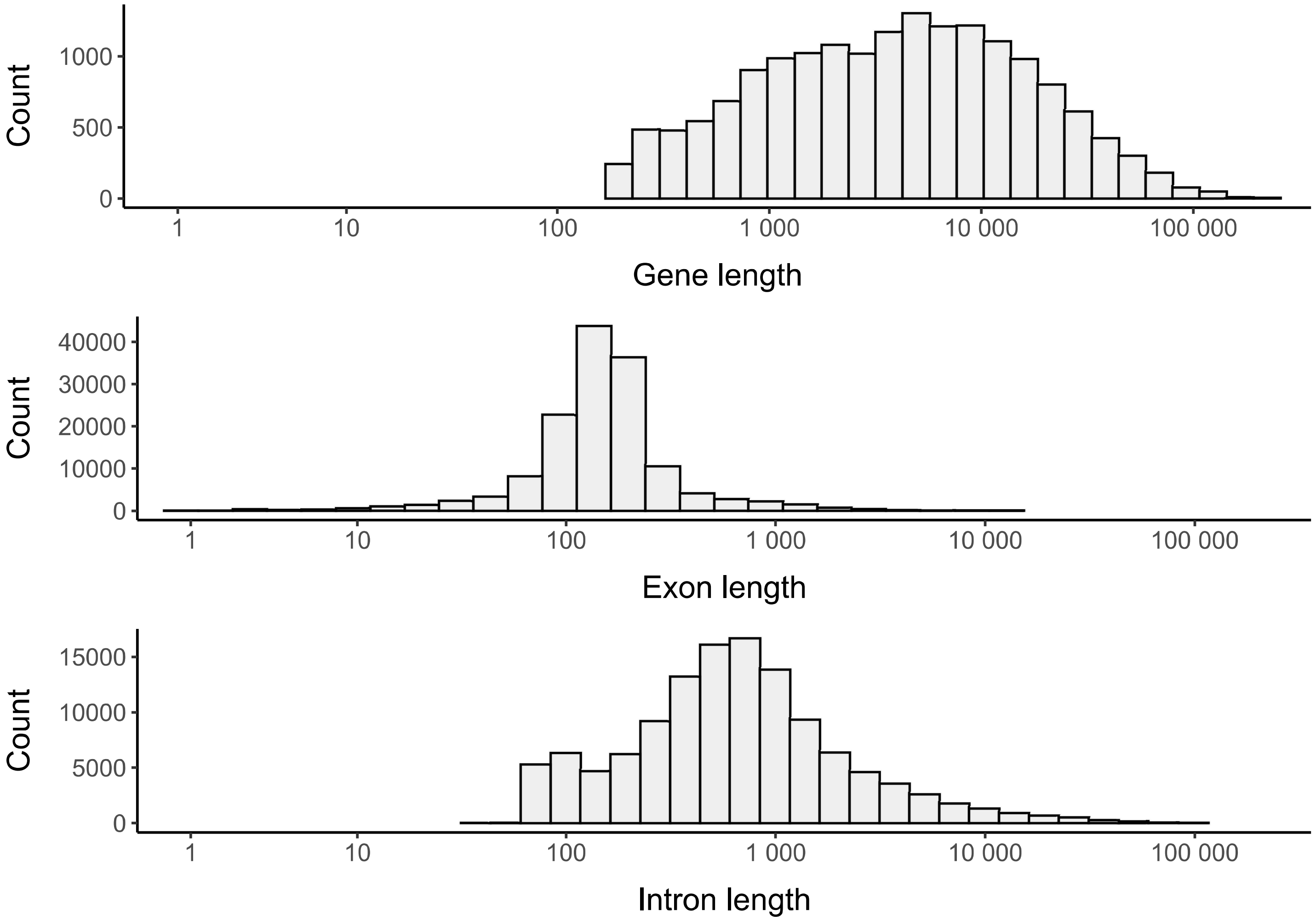

Figure S5 The distribution of gene, exon, and intron lengths, plotted on a log_{10} scale.

Table S1 Sampling locations and other metadata for *B. ino* samples used in this study.

Sample	Date	Sex	Locality	Region	Country	Lat	Long	Altitude	Collector	Data	Analyses
SO_BI_364	05/07/2017	Male	Somiedo, Braña de Mumian	Asturias	Spain	43.068	-6.24	1420	KL	Pacbio CLR + Illumina WGS	Contig assembly + Z identification
SO_BI_375	05/07/2017	Male	Somiedo, Braña de Mumian	Asturias	Spain	43.068	-6.24	1420	KL	Illumina HiC + Illumina WGS	Scaffolding + Karyotyping
SO_BI_376	05/07/2017	n/a	Somiedo, Braña de Mumian	Asturias	Spain	43.068	-6.24	1420	KL	Illumina RNA-seq	Gene annotation
FR_BI_1497 (RV-coll12O846)	11/08/2012	Female	Larche (Les Marmottes)	Alpes-de-Haute-Provence	France	44.446	6.851	1680	VD & Raluca Vodă	Illumina WGS	Z identification

Table S2 Annotated transposable elements

Repeat class	No. elements	Total length (Mb)	Percentage of genome (%)	No. distinct classifications
**Retroelement**	116256	47.36	11.49	930
SINE	26242	6.53	1.58	23
LINE	59472	25.92	6.29	611
Penelope	25145	6.89	1.67	34
LTR element	5397	8.02	1.95	262
**DNA transposon**	36793	11.48	2.79	598
**Rolling Circle**	258724	73.34	17.81	307
**Unclassified**	74790	23.74	5.76	339
**Other**	21	0.00	0.00	3
**Total**	486584	155.92	37.85	2177
